# Severe COVID-19-associated variants linked to chemokine receptor gene control in monocytes and macrophages

**DOI:** 10.1186/s13059-022-02669-z

**Published:** 2022-04-14

**Authors:** Bernard S. Stikker, Grégoire Stik, Antoinette F. van Ouwerkerk, Lianne Trap, Salvatore Spicuglia, Rudi W. Hendriks, Ralph Stadhouders

**Affiliations:** 1 Department of Pulmonary Medicine, Erasmus MC, University Medical Center Rotterdam, Rotterdam, The Netherlands; 2 Centre for Genomic Regulation (CRG) and Institute of Science and Technology (BIST), Barcelona, Spain; 3 Universitat Pompeu Fabra (UPF), Barcelona, Spain; 4 Aix-Marseille University, INSERM, TAGC, UMR 1090, Marseille, France; 5 Department of Cell Biology, Erasmus MC, University Medical Center Rotterdam, Rotterdam, The Netherlands

**Keywords:** SARS-CoV-2, COVID-19, 3p21.31, GWAS, Monocyte, Macrophage, Chemokine receptor, 3D genome organization, CTCF, Gene regulation

## Abstract

Genome-wide association studies have identified 3p21.31 as the main risk locus for severe COVID-19, although underlying mechanisms remain elusive. We perform an epigenomic dissection of 3p21.31, identifying a CTCF-dependent tissue-specific 3D regulatory chromatin hub that controls the activity of several chemokine receptor genes. Risk SNPs colocalize with regulatory elements and are linked to increased expression of *CCR1*, *CCR2* and *CCR5* in monocytes and macrophages. As excessive organ infiltration of inflammatory monocytes and macrophages is a hallmark of severe COVID-19, our findings provide a rationale for the genetic association of 3p21.31 variants with elevated risk of hospitalization upon SARS-CoV-2 infection.

**Supplementary Information:**

The online version contains supplementary material available at 10.1186/s13059-022-02669-z.

## Background

Coronavirus disease 2019 (COVID-19) is a potentially life-threatening respiratory disorder caused by the severe acute respiratory syndrome coronavirus 2 (SARS-CoV-2) [1]. Clinical manifestations of SARS-CoV-2 infection range from no or mild symptoms to respiratory failure. Life-threatening disease is often associated with an excessive inflammatory response to SARS-CoV-2, involving elevated systemic cytokine levels and profound organ infiltration by monocytes and macrophages [2, 3]. Besides clinical characteristics such as age and various comorbidities [4], genetic differences play a role in predisposing individuals to progress towards severe disease [5, 6]. In genome-wide association studies (GWASs), the 3p21.31 locus was strongly associated with increased risks of morbidity and mortality - in particular for younger (≤ 60 years) individuals [7]. However, it is currently still largely unclear how variants and genes in this locus affect the immune response against SARS-CoV-2 and COVID-19 disease pathophysiology.

## Results and discussion

**Fig. 1:**
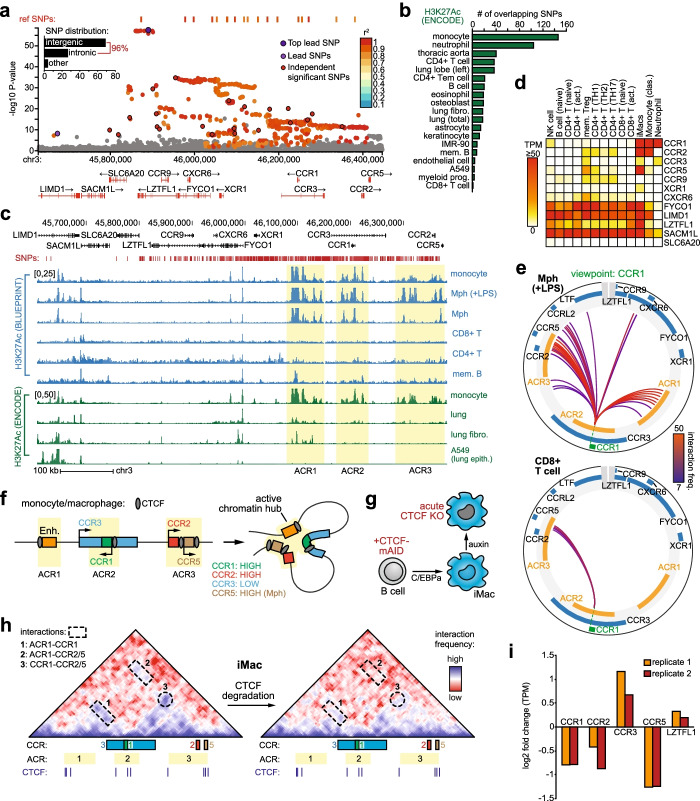
The 3p21.31 severe COVID-19 risk locus harbours a 3D chromatin hub that controls monocyte-macrophage chemokine receptor expression. **a** FUMA regional plot of the 3p21.31 locus highlighting all variants in high linkage-disequilibrium (*r*^2^>0.8, *P*<0.05) with independent significant (*P*<5e−8) GWAS SNPs. Bar graph denotes SNP distribution. **b** Number of COVID-19-associated SNPs overlapping with H3K27Ac^+^ regions in the indicated cell types. **c** UCSC genome browser view of H3K27Ac ChIP-Seq tracks for the indicated cell or tissue types (fibro. = fibroblast, epith. = epithelial, Mph = macrophage, mem. B = memory B cell). Genes and FUMA SNPs are shown above, yellow shading indicates location monocyte/macrophage-specific active chromatin regions (‘ACR1-3′). **d** Normalized gene expression levels (transcripts per million; TPM) of 3p21.31 candidate genes across various immune cell subsets from peripheral blood (DICE and HaemoSphere databases) and in vitro transdifferentiated induced macrophages (iMacs [10]). **e** Circos plots showing significant chromatin interactions with the *CCR1* promoter (green dashed line) in LPS stimulated macrophages or CD8+ T cells as measured by promoter-capture Hi-C (freq.: frequency). ACRs are indicated in orange. **f** Schematic indicating active chromatin hub formation involving the ACRs (enhancer; Enh.), CTCF binding sites and indicated *CCR* genes in monocytes/macrophages. **g** Experimental scheme depicting C/EBPα-driven transdifferentiation of B cells carrying CTCF-mAID alleles into iMacs. Exposure to auxin induces rapid degradation of CTCF-mAID [10]. **h** Hi-C interaction matrices (5 kb resolution, smoothened) for iMacs before (left) and after (right) auxin-inducible CTCF degradation, resulting in weaker interactions (indicated by numbers) between CCR genes and/or ACR1 (colour code as in panel f). CTCF ChIP-Seq peaks in iMacs are indicated below. **i** Gene expression changes of indicated genes in iMacs after CTCF degradation

COVID-19 GWAS meta-analyses (release 4 by the COVID-19 Host Genetics Initiative [8]) confirmed the strong association between the 3p21.31 locus and COVID-19, both when comparing hospitalized COVID-19 patients with healthy control subjects (Additional file 1: Fig.S1a) or with non-hospitalized patients (Additional file 1: Fig.S1b), indicating a stronger link with more severe disease. We focused on the former comparison (8638 hospitalized COVID-19 patients vs. 1,736,547 control subjects) to maximize the number of associated SNPs available for downstream analysis. Regional association plots generated using the Functional Mapping and Annotation (FUMA) platform [9] revealed a region of 743 kb with 21 independent significant (*P*<5e−8) GWAS SNPs and hundreds of variants in high linkage disequilibrium (LD; *r*^2^>0.8) (Fig. 1a). Approximately 96% of these SNPs fall in non-coding regions adjacent to 12 known protein-coding genes (Fig. 1a).

Common disease-associated genetic variants predominantly localize to regulatory DNA elements [11]. To identify disease-relevant candidate genes and gene regulatory regions at 3p21.31, we integrated GWAS findings with publicly available data from large-scale transcriptomics and epigenome profiling studies. Special emphasis was placed on immune cells, as detrimental hyperinflammation is characteristic of severe COVID-19 [2, 3]. Analysis of histone 3 lysine 27 acetylation (H3K27Ac) profiles from ENCODE [12] and BLUEPRINT [13] databases revealed cell type-specific active gene regulatory elements (GREs) at 3p21.31, with particularly strong activity seen in monocytes, monocyte-derived macrophages and neutrophils (Fig. 1b, c, Additional file 1: Fig.S2). The largest fraction of disease-associated SNPs overlapped with monocyte H3K27Ac^+^ GREs, which were concentrated in three active chromatin regions (ACRs) near the *CCR1*, *CCR2*, *CCR3* and *CCR5* genes (Fig. 1b, c). CCR1 and CCR2 are critical mediators of monocyte/macrophage polarization and tissue infiltration [14], which are pathogenic hallmarks of severe COVID-19 [2, 3]. The three ACRs also showed substantial chromatin accessibility (as measured by DNAse-Seq) in monocytes (Additional file 1: Fig.S2). Gene expression analysis using data from 6 transcriptome repositories (see the “Methods” section) confirmed strong transcriptional activity of the 3′ *CCR* genes in tissues containing haematopoietic cells (e.g. whole blood, spleen), with especially *CCR1* and *CCR2* being highly expressed in classical monocytes, macrophages and neutrophils (Fig. 1d, Additional file 1: Figs. S2-S3). Of note, several other immune cell subsets, including T cell and dendritic cell subsets, also expressed specific *CCR* genes (Fig. 1d, Additional file 1: Figs. S2-S3). Chromatin interaction profiles from primary immune cells (measured by promoter-capture Hi-C [15]) revealed extensive monocyte/macrophage-specific chromatin interactions between the three ACRs, as exemplified by *CCR1* promoter interaction profiles in monocyte-derived macrophages and T cells (Fig. 1e, Additional file 1: Fig.S4a-b). In all immune cells profiled by Javierre et al. [15], no significant interactions were detected between 3p21.31 gene promoters and the lead SNP region or the most distal SNPs in *LIMD1* (Additional file 1: Fig.S4c), although HindIII-based promoter-capture Hi-C has limited resolution very close (<20 kb) to viewpoints.

Together, this analysis reveals the strong transcriptional activity of a *CCR* gene cluster within the 3p21.31 COVID-19 risk locus in immune cells, especially in monocytes and macrophages. Activity is centred around *CCR1* and its genomic surroundings, which are organized in a 3D chromatin hub involving the other active *CCR* genes (i.e. *CCR2*, *CCR5*) and putative enhancer elements (Fig. 1f)—a chromatin conformation often used for complex tissue-specific gene regulation [16]. To further substantiate the relevance of local 3D chromatin organization for 3p21.31 *CCR* gene regulation in myeloid cells, we used epigenomics data from the BLaER induced macrophage (iMac) cell line system [10]. The iMacs, which morphologically and functionally closely resemble macrophages [17], showed highly comparable H3K27Ac enrichment at the 3p21.31 ACRs and expressed high levels of *CCR1*, *CCR2* and *CCR5* (Additional file 1: Fig.S5a-b). High-resolution in-situ Hi-C data [10] of iMacs revealed that the 3p21.31 COVID-19-associated genomic block resides in the nuclear A compartment (Additional file 1: Fig.S5c), a chromosomal compartment located in the nuclear interior that groups together transcriptionally active chromatin [18]. Zooming in, we observed that most of the 3p21.31 risk variants and all associated chemokine receptor genes localize to a single topologically associating domain (TAD) (Additional file 1: Fig.S5d), representing an insulated genomic neighbourhood that promotes establishing interactions between genes and regulatory elements inside the TAD [18]. Interestingly, ACR1 and ACR3 were flanked by strong binding sites for the genome architectural CCCTC-binding factor CTCF [19] in iMacs and primary monocytes (Additional file 1: Fig.S5a). Together with the presence of additional CTCF binding sites within all three ACRs, including the CCR1 promoter region (Additional file 1: Fig.S5a), these data suggest that CTCF organizes local 3D active chromatin hub formation to insulate the *CCR3*-*CCR1*-*CCR2*-*CCR5* gene cluster for transcriptional regulation. To test this hypothesis, we leveraged our recently developed iMac line expressing CTCF fused to an auxin-inducible degron (mAID), which allows for rapid degradation of CTCF and disruption of 3D genome architecture (Fig. 1g) [10]. Detailed Hi-C analysis confirmed the presence of strong interactions between the ACRs and 3′ *CCR* genes in iMacs, which were disrupted upon CTCF depletion (Fig. 1h). Importantly, chromatin hub decommissioning specifically reduced *CCR1*, *CCR2* and *CCR5* expression (Fig. 1i), revealing that CTCF-mediated 3D chromatin interactions are critical for regulating 3p21.31 *CCR* gene activity in macrophages. Of note, expression of the *CCRL2* gene just downstream of *CCR5*—encoding an atypical chemokine receptor involved in macrophage polarization [20]—was only marginally affected by CTCF depletion (log_2_ fold change of 0.23).

**Fig. 2:**
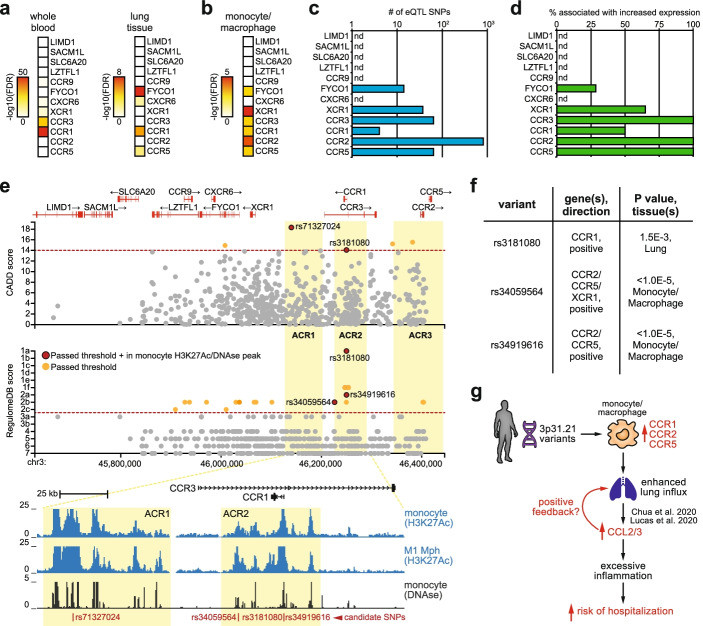
3p21.31 COVID-19 risk variants are linked to increased monocyte-macrophage chemokine receptor gene expression. **a**, **b** Heatmap depicting statistical association strength (using adjusted *P* values) for the peak eQTL SNP in whole blood and lung tissue (panel **a**, GTEx v8) or in monocytes and macrophages (panel **b**, see the “Methods” section for eQTL sources). Only variants in high linkage-disequilibrium (*r*^2^>0.8, *P*<0.05) with independent significant (*P* <5e−8) GWAS SNPs were considered. **c** Number of eQTL SNPs associated with the indicated 3p21.31 genes in monocytes and macrophages. **d** Percentage of eQTL SNPs associated with increased expression of the indicated 3p21.31 genes in monocytes and macrophages. **e** CADD and RegulomeDB scores for all FUMA SNPs across the 3p21.31 locus. Red dashed lines indicate selected thresholds. SNPs passing the threshold are indicated in orange; those within monocyte H3K27Ac+/DNAse+ regulatory regions are in red. Below is depicted a UCSC genome browser view of H3K27Ac ChIP-Seq and DNAse-seq signals in the indicated cell types centred on the four candidate causal variants in ACR1 and ACR2. **f** eQTL analysis showing the three candidate causal variants that are also eQTLs for CCR1, CCR2 and CCR5. Direction of the association, *P* values and tissue/cell types are indicated. **g** Schematic indicating how 3p21.31 variants may increase risk of severe disease upon SARS-CoV-2 infection through altered monocyte-macrophage chemotactic receptor expression. See text for details

We next sought to directly link COVID-19-associated genetic variants to altered 3′ *CCR* gene expression in myeloid immune cells. To this end, we used FUMA to systematically analyze previously reported expression quantitative trait loci (eQTLs) overlapping with the 958 COVID-19-associated (*P*<5e−8) SNPs. As eQTL sources, we focused on disease-relevant tissues rich in monocytes/macrophages (i.e. whole blood and lung tissue) and studies using purified monocytes or in vitro differentiated macrophages (see the “Methods” section). The 3′ 3p21.31 *CCR* genes showed highly significant eQTL associations (FDR <0.05) with COVID-19-associated variants, especially in monocytes and macrophages (Fig. 2a, b). Multiple risk SNPs were identified as eQTLs for *CCR1*, *CCR2*, *CCR3* and *CCR5* in monocytes/macrophages, with the majority correlating with increased gene expression (Fig. 2c, d). No eQTL associations were detected for *CCRL2*. To further prioritize variants with potential biological significance we used RegulomeDB [21] and CADD [22] SNP annotations. Stringent filters for both scores were combined with localization within a putative monocyte regulatory region (H3K27Ac^+^ and DNAse^+^), yielding four unique candidate causal SNPs of which three were associated with increased *CCR1*, *CCR2* and/or *CCR5* expression (Fig. 2e, f). These variants did not engage in significant interactions with sequences far outside the susceptibility region, e.g. beyond the CCR gene cluster (Additional file 1: Fig.S6). Candidate causal variants mostly clustered within ACR2 and altered putative transcription factor binding motifs, readily providing testable hypotheses for future investigations (Additional file 1: Fig.S7). For example, two SNPs within the *CCR1* promoter affected binding motifs of known regulators of the macrophage inflammatory expression programme (Additional file 1: Fig.S7a-b). Variant rs3181080 optimizes a composite Interferon Regulatory Factor (IRF)-Activator Protein 1 (AP1) motif, which is used for cooperative binding of IRF and AP1 family transcription factors that promote monocyte/macrophage activation [23]. In line with *CCR1* activation by IRF/AP1 factors, binding of AP1 proteins and IRF4 to rs3181080 was detected in *CCR1*-expressing GM12878 lymphoblastoid cells (Additional File 1: Fig.S7c). Previous experiments in mouse macrophages [24] confirmed IRF binding to the *Ccr1* promoter (Additional file 1: Fig.S7d). The second *CCR1* promoter variant, rs34919616, disrupts a critical nucleotide in a motif for BCL6 (Additional file 1: Fig.S7a-b), a suppressor of inflammatory gene expression in macrophages [25].

Taken together, these data show that the COVID-19-associated 3p21.31 locus harbours a CTCF-dependent tissue-specific 3D chromatin hub that controls chemotactic receptor expression in monocytes and macrophages. Several 3p21.31 variants localize to gene regulatory elements within this chromatin hub and are associated with elevated *CCR1*, *CCR2*, *CCR3* and *CCR5* expression, which is further supported by a recent transcriptome-wide association study in lung tissue [6]. Mechanistically, these risk variants may modulate transcription factor binding at *CCR* gene regulatory elements. CCR1, CCR2 and CCR5 upregulation could enhance lung infiltration by monocytes and macrophages upon viral infection [14], contributing to the rapid and deleterious hyperinflammation observed in COVID-19 patients suffering from severe disease [2, 3] (Fig. 2g). In support of this notion, single cell transcriptomics revealed increased levels of *CCR1* and *CCR5* as well as their ligands *CCL2*/*CCL3* specifically in pulmonary macrophages from critical COVID-19 patients [26, 27]. Additionally, CCL2 plasma levels showed the highest predictive value for mortality in a COVID-19 patient cohort [28]. These findings are in line with excessive pulmonary influx of monocytes and subsequent differentiation into inflammatory tissue macrophages as a hallmark of severe COVID-19 (Fig. 2g) [26].

Our analysis has several limitations. Although we provide compelling evidence for monocyte-macrophage 3’ *CCR* gene activity linked to 3p21.31 risk variants, several other immune cell types involved in antiviral immunity also express some of these chemokine receptors (e.g. *CCR1* on neutrophils, *CCR5* on T cell subsets) and may therefore also be affected by the genetic variants. Moreover, although our analysis detected fewer non-coding regulatory activity in the 5′ part of the 3p21.31 COVID-19-associated genomic block, this region harbours the lead SNP and several actively transcribed genes with more housekeeping-like expression patterns, which may also be relevant for COVID-19 pathophysiology. Indeed, Downes et al. recently reported that a variant in high LD with the lead SNPs affects an enhancer of *LZTFL1* in non-immune cells, with potential implications for anti-viral responses [29]. Although variants in the lead SNP region were also reported as eQTLs for *CCR2* and *CCR5* in monocytes and macrophages, this likely reflects the high LD (*r*^2^>0.8) of these variants with the 3′ *CCR* SNPs (Fig. 1a). In support of this notion, genetic deletion of a 68kb region around the lead SNP in a myeloid cell line did not affect 3′ 3p21.31 *CCR* gene expression [30] and Downes et al. found no evidence of these SNPs disrupting gene regulatory mechanisms in immune cells [29]. Another study integrating loss-of-function experiments in an airway epithelial carcinoma cell line with eQTL data implicated *SLC6A20* and *CXCR6* in COVID-19 pathophysiology [31], whereas deleting the lead SNP region resulted in reduced *CCR9* and *SLC6A20* expression in leukemic T cells [30]. Future investigations including additional (non-immune) cell types are required to further elucidate the candidate causal genes operating in different cell types and/or under different microenvironmental circumstances.

## Conclusions

Our data support a scenario in which common genetic variants increase susceptibility to develop severe COVID-19 by affecting gene regulatory control of monocyte-macrophage chemotactic receptor expression. As a consequence, elevated migratory capacity of monocytes and macrophages could contribute to aggravated inflammatory responses and more severe disease. These data add to our understanding of the genetic basis of COVID-19 disease heterogeneity and support exploring therapeutic targeting of monocyte-macrophage 3p21.31 CCR activity in hospitalized COVID-19 patients.

## Methods

### GWAS data retrieval

Version 4 COVID-19 GWAS meta-analysis data was retrieved from The COVID-19 Host Genetics Initiative at https://www.covid19hg.org/. GWAS data (GRCh37/hg38 genome build) was obtained from two studies: B1_ALL (hospitalized COVID-19 vs. non-hospitalized COVID-19; 2430 cases versus 8478 controls) and B2_ALL (hospitalized COVID-19 vs. population; 8638 cases versus 1,736,547 controls). GWAS summary statistics files were used to generate input files for FUMA using standard data frame processing functions in Rstudio v.1.3.

### Identification of a high LD block of COVID-19 associated SNPs

FUMA [9] was performed for both B1_ALL and B2_ALL GWASs (version 4 summary statistics downloaded from https://www.covid19hg.org/) using default settings, with exception of the *r*^2^ (LD) used to define independent significant SNPs, which was set to ≥0.8. Manhattan and regional plots were generated by FUMA’s SNP2GENE function. Significant FUMA SNPs were converted to GRCh38/hg38 using UCSC LiftOver (https://genome.ucsc.edu/cgi-bin/hgLiftOver) to allow aligning variants to the epigenomic profiles.

### ChIP-Seq, DNAse-Seq and (promoter-capture) Hi-C data analysis

ChIP-Seq and DNAse-Seq epigenomic data used were retrieved from public ENCODE [12] and BLUEPRINT [13] databases. Data were visualized in the UCSC Genome Browser (https://genome.ucsc.edu). The *intersect* function of BEDTools [32] was used to determine the number of FUMA SNPs overlapping with H3K27Ac^+^ regions in the indicated cell types. Peak calling files for each H3K27Ac dataset were directly obtained from the ENCODE website (https://www.encodeproject.org/). Circos plots visualizing promoter-capture HiC data from the BLUEPRINT consortium [15] were generated using https://www.chicp.org/chicp/, with a threshold normalized interaction value of 7. ChIP-Seq and in-situ Hi-C data from in vitro transdifferentiated macrophages (induced macrophages or iMacs), both prior to and after auxin-inducible CTCF degradation, were obtained from GSE140528 and analysed as previously described [10].

### Gene expression analysis

RNA-Seq profiles from a broad spectrum of selected relevant cell types were obtained from public ENCODE [12] and BLUEPRINT [13] databases and visualized in the UCSC Genome Browser. Expression value heatmaps from various collection of (immune) cell types were obtained from DICE [33] (https://dice-database.org/), GTEx v8 [34] (via FUMA’s GENE2FUNCTION function), BioGPS [35] (http://biogps.org/), Haemosphere [36] (https://www.haemosphere.org/) and Monaco et al. [37] (GSE107011). Transcripts per million (TPM) values were visualized as averaged values using Morpheus (https://software.broadinstitute.org/morpheus/). RNA-Seq and TPM values for iMacs were obtained from GSE140528 and analysed as previously described [10].

### Candidate causal variant filtering

The Combined Annotation Dependent Depletion (CADD [22]) and RegulomeDB [21] scores for all significantly associated SNPs were also obtained from FUMA. As thresholds to identify candidate causal variants, we used CADD scores >14 and RegulomeDB scores <3. SNPs were further filtered based on their combined overlap with H3K27Ac ChIP-Seq and DNAse-seq peaks in monocytes (data obtained from ENCODE [12]). Transcription factor binding motifs were obtained using HOMER [38].

### Expression quantitative trait locus (eQTL) analysis

eQTL analysis was performed using FUMA, focusing on tissues relevant for COVID-19 pathophysiology and enriched for monocytes/macrophages (i.e. whole blood and lung from GTEx v8 [34]) or studies using monocytes and/or *in vitro* differentiated macrophages [39–41]. Thresholds for statistical significance were set to FDR<0.05.

## Supplementary Information

**Additional file 1: Figure S1.** Overview of genome-wide genetic associations with severe COVID-19. **Figure S2.** Transcriptional and epigenomic activity at 3p21.31 in selected cell types. **Figure S3.** Gene expression analysis of 3p21.31 candidate genes across various nonimmune and immune cells types. **Figure S4.** Regulatory chromatin interactions across the 3p21.31 COVID-19 risk locus. **Figure S5.** Epigenomic landscape and 3D genome folding at the 3p21.31 COVID-19 risk locus in iMacs. **Figure S6.** Chromatin interactions with prioritized 3p21.31 COVID-19 risk variants. **Figure S7.** 3p21.31 COVID-19 risk variants disrupt putative transcription factor binding sites.

**Additional file 2: Table S1.** Database accession numbers of individual datasets used and accompanying citations used throughout this study.

**Additional file 3.** Review history.

## Data Availability

All datasets generated and/or analysed during the current study are available in the public repositories and/or using the persistent web links (also see Methods section): GWAS data was retrieved from https://www.covid19hg.org/ (version 4, B1_ALL & B2_ALL); FUMA analysis (including eQTL analysis) was performed using https://fuma.ctglab.nl/; ChIP-Seq, DNAse-Seq and RNA-Seq data used were retrieved from public ENCODE (https://www.encodeproject.org/), BLUEPRINT (http://dcc.blueprint-epigenome.eu/#/home), DICE (https://dice-database.org/), GTEx (https://gtexportal.org/home/), BioGPS (http://biogps.org/) and Haemosphere (https://www.haemosphere.org/) databases; epigenomics data was visualized in the UCSC Genome Browser (https://genome.ucsc.edu); promoter-capture Hi-C data from the BLUEPRINT consortium was visualized using https://www.chicp.org/chicp/; iMac Hi-C and RNA-Seq data was obtained from GSE140528; heatmaps were generated using Morpheus (https://software.broadinstitute.org/morpheus/). Database accession numbers of individual datasets used and accompanying citations can be found in Additional file 2: Table S1.
