# Supplementary material for: Severe COVID-19-associated variants linked to chemokine receptor gene control in monocytes and macrophages
Source: Genome Biol. 2022 Apr 14;23:96. doi: 10.1186/s13059-022-02669-z (PMC9009160; doi:10.1186/s13059-022-02669-z)
Supplement: Supplementary file 1 — Additional file 1: Figure S1. Overview of genome-wide genetic associations with severe COVID-19. Figure S2. Transcriptional and epigenomic activity at 3p21.31 in selected cell types. Figure S3. Gene expression analysis of 3p21.31 candidate genes across various nonimmune and immune cells types. Figure S4. Regulatory chromatin interactions across the 3p21.31 COVID-19 risk locus. Figure S5. Epigenomic landscape and 3D genome folding at the 3p21.31 COVID-19 risk locus in iMacs. Figure S6. Chromatin interactions with prioritized 3p21.31 COVID-19 risk variants. Figure S7. 3p21.31 COVID-19 risk variants disrupt putative transcription factor binding sites. [file 13059_2022_2669_MOESM1_ESM.pdf]

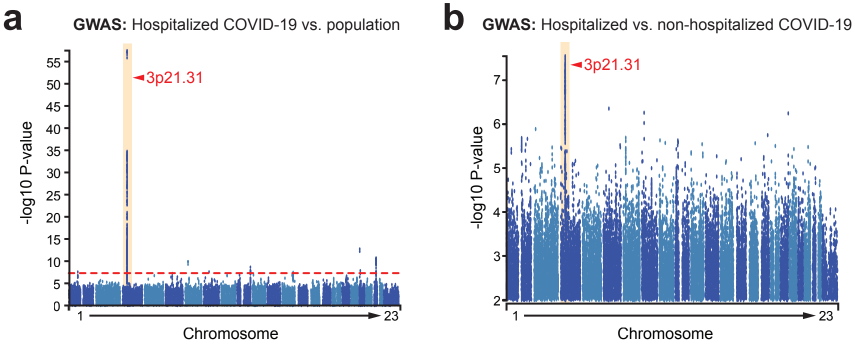
a
GWAS: Hospitalized COVID-19 vs. population
-log10 P-value
55
50
45
40
35
30
25
20
15
10
5
0
3p21.31
1
23
Chromosome
b
GWAS: Hospitalized vs. non-hospitalized COVID-19
-log10 P-value
7
6
5
4
3
2
3p21.31
1
23
Chromosome

Figure S1

Figure S1. Overview of genome-wide genetic associations with severe COVID-19. (**a,b**) Manhattan plots showing the strong association of the 3p21.31 locus with hospitalized COVID-19 (based on v4 GWAS meta-analysis release). Panel a compares hospitalized COVID-19 patients with healthy control subjects; panel b compares hospitalized with non-hospitalized patients.

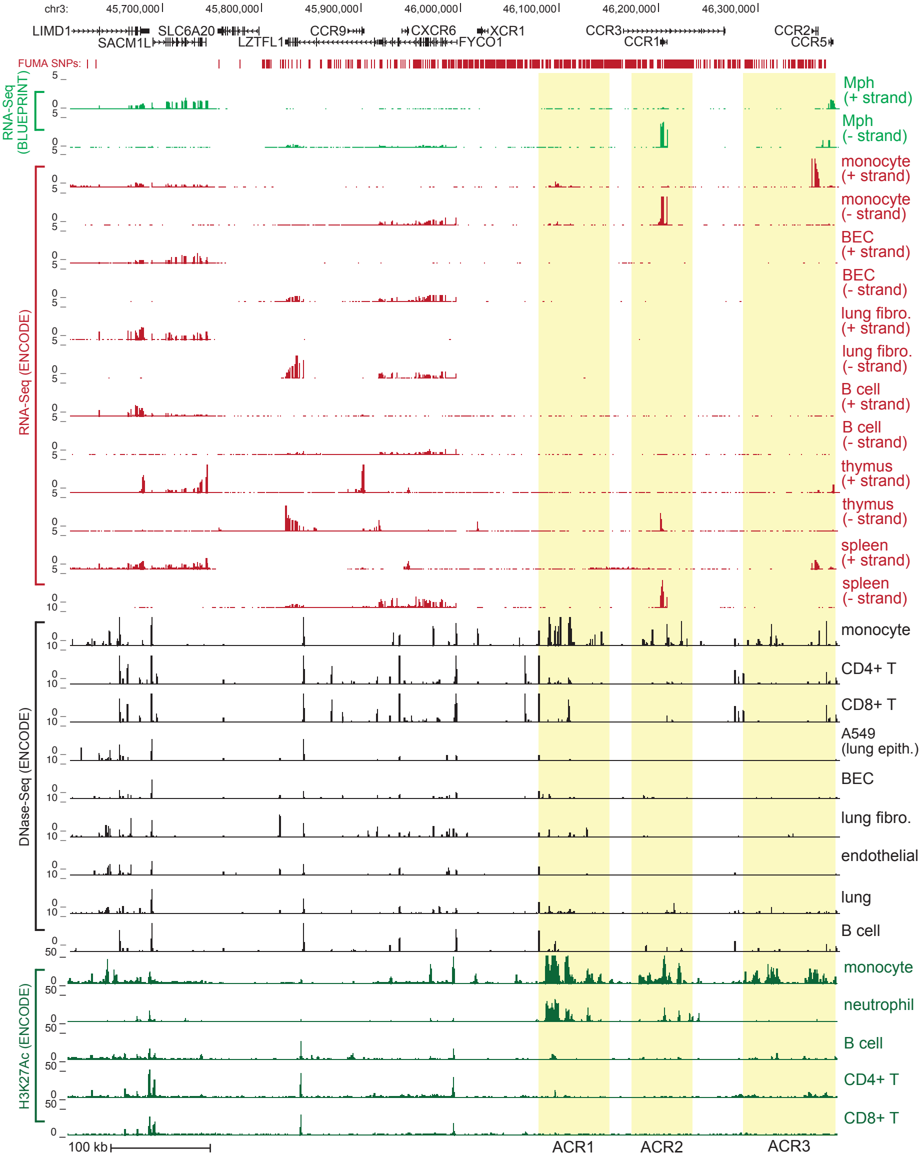

chr3:
45,700,000
45,800,000
45,900,000
46,000,000
46,100,000
46,200,000
46,300,000
LIMD1
SACM1L
SLC6A20
LZTFL1
CCR9
CXCR6
FYCO1
XCR1
CCR3
CCR1
CCR2
CCR5
FUMA SNPs:
RNA-Seq (BLUEPRINT)
Mph (+ strand)
Mph (- strand)
RNA-Seq (ENCODE)
monocyte (+ strand)
monocyte (- strand)
BEC (+ strand)
BEC (- strand)
lung fibro. (+ strand)
lung fibro. (- strand)
B cell (+ strand)
B cell (- strand)
thymus (+ strand)
thymus (- strand)
spleen (+ strand)
spleen (- strand)
DNase-Seq (ENCODE)
monocyte
CD4+ T
CD8+ T
A549 (lung epith.)
BEC
lung fibro.
endothelial
lung
B cell
H3K27Ac (ENCODE)
monocyte
neutrophil
B cell
CD4+ T
CD8+ T
100 kb
ACR1
ACR2
ACR3

Figure S2

Figure S2. Transcriptional and epigenomic activity at 3p21.31 in selected cell types. UCSC genome browser overview of RNA-Seq, DNAse-Seq and H3K27Ac ChIP-Seq tracks for the indicated cell or tissue types (BEC: bronchial epithelial cell, fibro.: fibroblast, epith.: epithelial). Yellow shading indicates location of monocyte/macrophage-specific active chromatin regions (‘ACR1-3’).

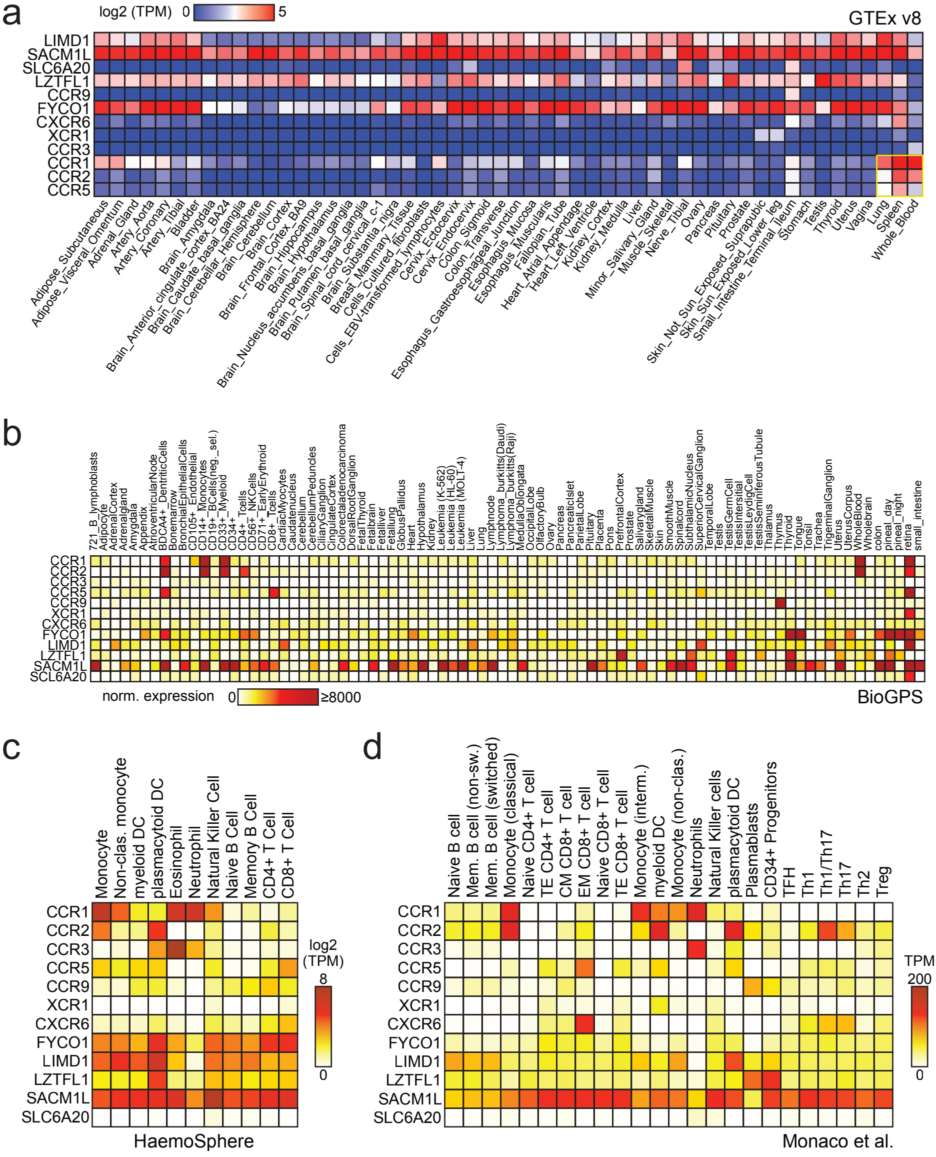

a
log2 (TPM) 0 5
GTEx v8
LIMD1
SACM1L
SLC6A20
LZTFL1
CCR9
FYCO1
CXCR6
XCR1
CCR3
CCR1
CCR2
CCR5
Adipose_Subcutaneous
Adipose_Visceral_Omentum
Adrenal_Gland
Artery_Aorta
Artery_Coronary
Artery_Tibial
Bladder
Brain_Amygdala
Brain_Anterior_cingulate_cortex_BA24
Brain_Caudate_basal_ganglia
Brain_Cerebellar_Hemisphere
Brain_Cerebellum
Brain_Cortex
Brain_Frontal_Cortex_BA9
Brain_Hippocampus
Brain_Hypothalamus
Brain_Nucleus_accumbens_basal_ganglia
Brain_Putamen_basal_ganglia
Brain_Spinal_cord_cervical_c-1
Brain_Substantia_nigra
Breast_Mammary_Tissue
Cells_Cultured_fibroblasts
Cells_EBV-transformed_lymphocytes
Cervix_Ectocervix
Cervix_Endocervix
Colon_Sigmoid
Colon_Transverse
Esophagus_Gastroesophageal_Junction
Esophagus_Mucosa
Esophagus_Muscularis
Fallopian_Tube
Heart_Atrial_Appendage
Heart_Left_Ventricle
Kidney_Cortex
Kidney_Medulla
Liver
Minor_Salivary_Gland
Muscle_Skeletal
Nerve_Tibial
Ovary
Pancreas
Pituitary
Prostate
Skin_Not_Sun_Exposed_Suprapubic
Skin_Sun_Exposed_Lower_leg
Small_Intestine_Terminal_Ileum
Stomach
Testis
Thyroid
Uterus
Vagina
Lung
Spleen
Whole_Blood
b
721_B_lymphoblasts
Adipocyte
AdrenalCortex
Adrenalgland
Amygdala
Appendix
AtrioventricularNode
BDCA4+_DentriticCells
Bonemarrow
BronchialEpithelialCells
CD105+_Endothelial
CD14+_Monocytes
CD19+_BCells(neg._sel.)
CD33+_Myeloid
CD34+
CD4+_Tcells
CD56+_NKCells
CD71+_EarlyErythroid
CD8+_Tcells
CardiacMyocytes
Caudatenucleus
Cerebellum
CerebellumPeduncles
CiliaryGanglion
CingulateCortex
Colorectaladenocarcinoma
DorsalRootGanglion
FetalThyroid
Fetalbrain
Fetalliver
Fetallung
GlobusPallidus
Heart
Hypothalamus
Kidney
Leukemia (K-562)
Leukemia (HL-60)
Leukemia (MOLT-4)
Liver
Lung
Lymphnode
Lymphoma_burkitts(Daudi)
Lymphoma_burkitts(Raji)
MedullaOblongata
OccipitalLobe
OlfactoryBulb
Ovary
Pancreas
PancreaticIslet
ParietalLobe
Pituitary
Placenta
Pons
PrefrontalCortex
Prostate
Salivarygland
SkeletalMuscle
Skin
SmoothMuscle
Spinalcord
SubthalamicNucleus
SuperiorCervicalGanglion
TemporalLobe
Testis
TestisGermCell
TestisIntersitial
TestisLeydigCell
TestisSeminiferousTubule
Thalamus
Thymus
Thyroid
Tongue
Tonsil
Trachea
TrigeminalGanglion
Uterus
UterusCorpus
WholeBlood
Wholebrain
colon
pineal_day
pineal_night
retina
small_intestine
CCR1
CCR2
CCR3
CCR5
CCR9
XCR1
CXCR6
FYCO1
LIMD1
LZTFL1
SACM1L
SCL6A20
norm. expression 0 ≥8000
BioGPS
c
Monocyte
Non-clas. monocyte
myeloid DC
plasmacytoid DC
Eosinophil
Neutrophil
Natural Killer Cell
Naive B Cell
Memory B Cell
CD4+ T Cell
CD8+ T Cell
CCR1
CCR2
CCR3
CCR5
CCR9
XCR1
CXCR6
FYCO1
LIMD1
LZTFL1
SACM1L
SLC6A20
log2 (TPM)
8
0
HaemoSphere
d
Naive B cell
Mem. B cell (non-sw.)
Mem. B cell (switched)
Monocyte (classical)
Naive CD4+ T cell
TE CD4+ T cell
CM CD8+ T cell
EM CD8+ T cell
Naive CD8+ T cell
TE CD8+ T cell
Monocyte (interm.)
myeloid DC
Monocyte (non-clas.)
Neutrophils
Natural Killer cells
plasmacytoid DC
Plasmablasts
CD34+ Progenitors
TFH
Th1
Th1/Th17
Th17
Th2
Treg
CCR1
CCR2
CCR3
CCR5
CCR9
XCR1
CXCR6
FYCO1
LIMD1
LZTFL1
SACM1L
SLC6A20
TPM
200
0
Monaco et al.

Figure S3

Figure S3. Gene expression analysis of 3p21.31 candidate genes across various non-immune and immune cells types. (**a**) Normalized gene expression levels (transcripts per million; TPM) across 54 tissues (GTEx v8 database). (**b**) Normalized gene expression levels (microarray-derived) across 84 tissues and cell types (BioGPS database). (**c**) Normalized gene expression levels (TPM) across 11 immune cell types (HaemoSphere). (**d**) Normalized gene expression levels (TPM) across 24 immune cell types (Monaco et al.[37]).

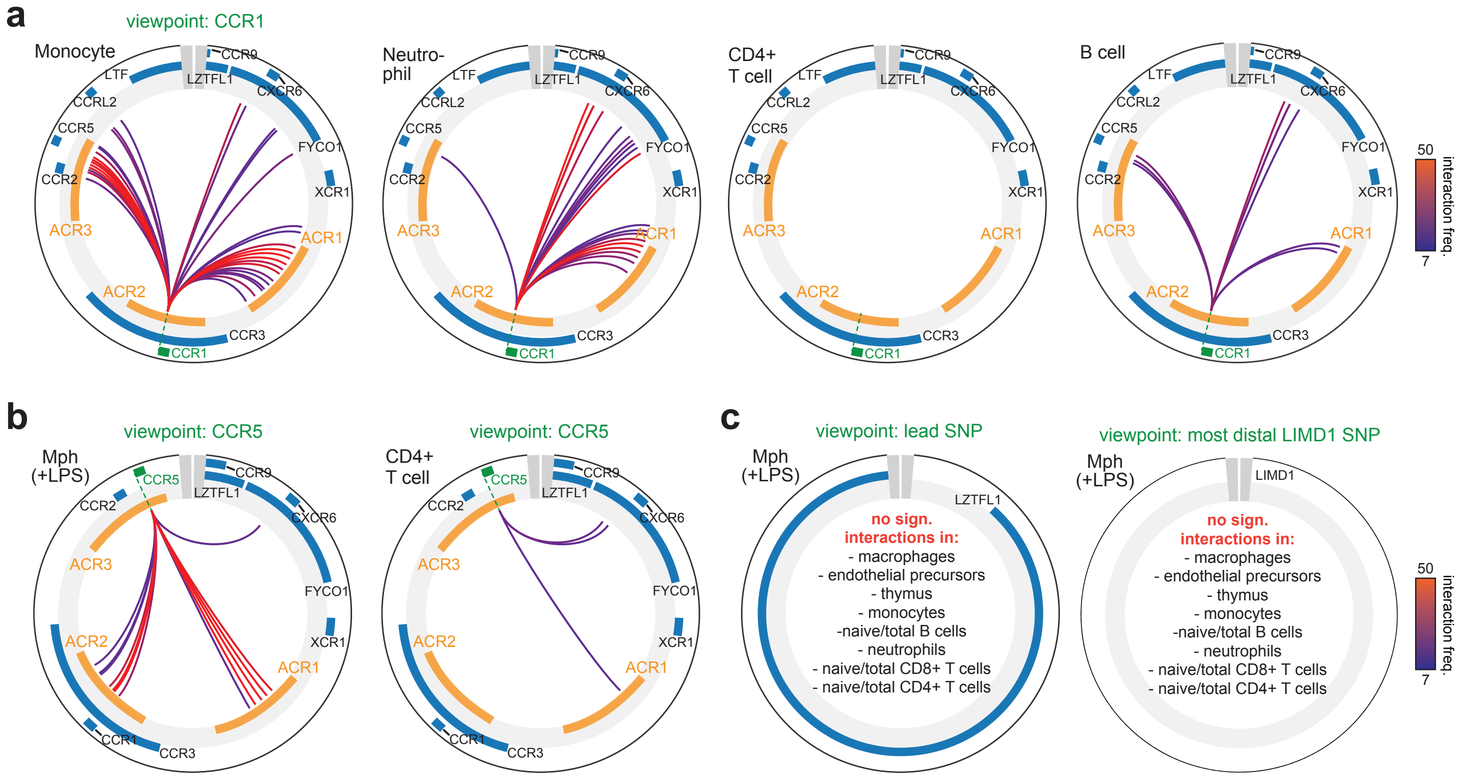
a
viewpoint: CCR1
Monocyte
Neutro-phil
CD4+ T cell
B cell
LTF
LZTFL1
CCR9
CXCR6
CCRL2
CCR5
FYCO1
CCR2
XCR1
ACR3
ACR1
ACR2
CCR3
CCR1
50
7
interaction freq.
b
viewpoint: CCR5
Mph (+LPS)
CD4+ T cell
c
viewpoint: lead SNP
viewpoint: most distal LIMD1 SNP
LIMD1
no sign. interactions in:
- macrophages
- endothelial precursors
- thymus
- monocytes
-naive/total B cells
- neutrophils
- naive/total CD8+ T cells
- naive/total CD4+ T cells

Figure S4

Figure S4. Regulatory chromatin interactions across the 3p21.31 COVID-19 risk locus. (**a**) Circos plots showing significant chromatin interactions with the CCR1 promoter (green dashed line) in monocytes, neutrophils, CD4+ T cells and total B cells measured by promoter-capture Hi-C[14] (freq.: frequency). ACRs are indicated in orange. (**b**) Significant chromatin interactions with the CCR5 promoter (green dashed line) in LPS stimulated macrophages (Mph+LPS) or CD4+ T cells. (**c**) Lack of chromatin interactions emanating from the location of the lead GWAS SNP (left circos plot) or most distal SNP in the LIMD1 gene (right circos plot). Data shown is for LPS stimulated macrophages (Mph+LPS, but identical results were obtained for the indicated cell types.

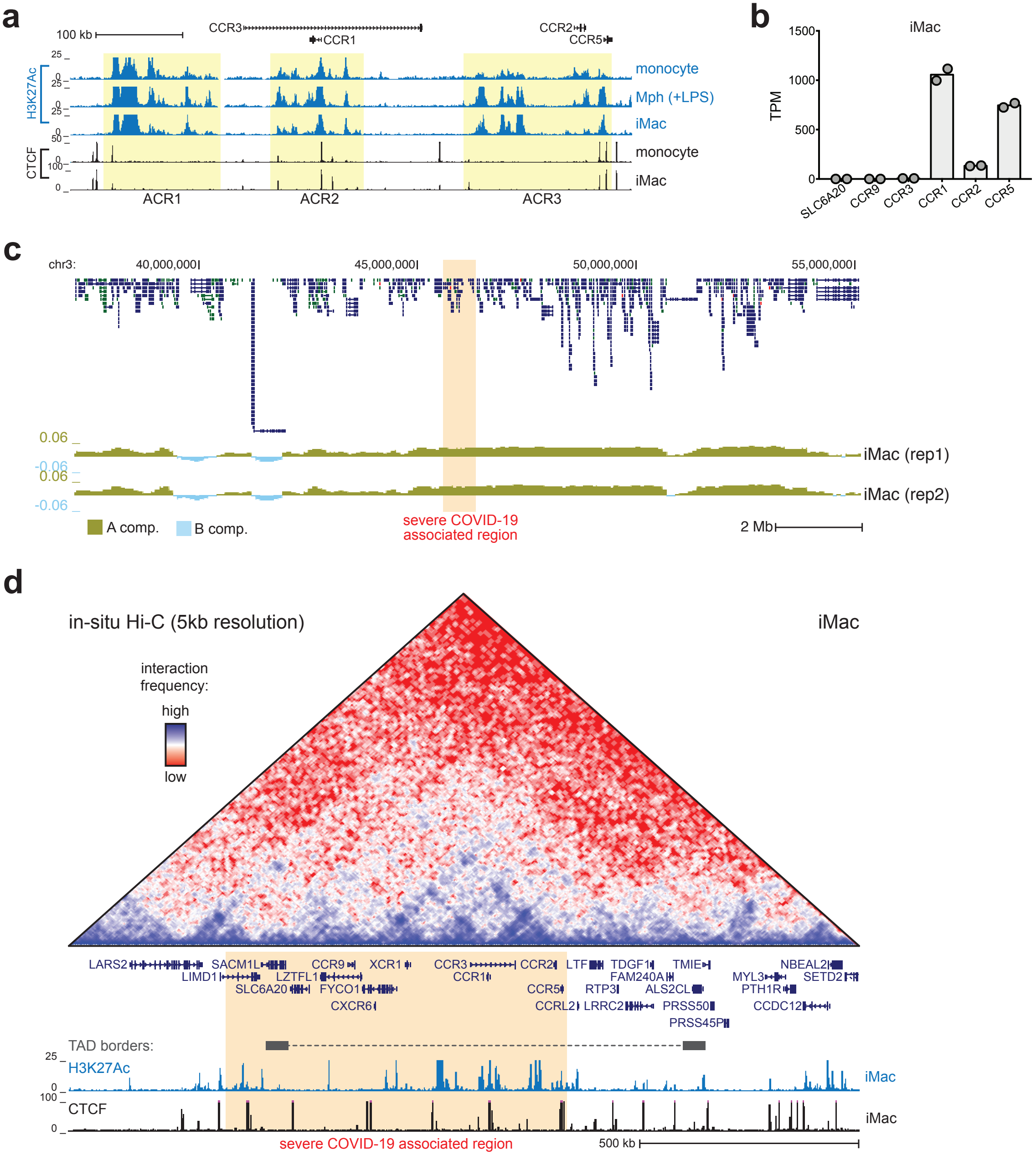
a
100 kb
CCR3
CCR1
CCR2
CCR5
H3K27Ac
monocyte
Mph (+LPS)
iMac
CTCF
monocyte
iMac
ACR1
ACR2
ACR3
b
iMac
TPM
SLC6A20
CCR9
CCR3
CCR1
CCR2
CCR5
c
chr3:
40,000,000
45,000,000
50,000,000
55,000,000
iMac (rep1)
iMac (rep2)
A comp.
B comp.
severe COVID-19 associated region
2 Mb
d
in-situ Hi-C (5kb resolution)
iMac
interaction frequency:
high
low
LARS2
SACM1L
CCR9
XCR1
CCR3
CCR2
LTF
TDGF1
TMIE
NBEAL2
LIMD1
LZTFL1
CCR1
FAM240A
MYL3
SETD2
SLC6A20
FYCO1
CCR5
RTP3
ALS2CL
PTH1R
CXCR6
CCRL2
LRRC2
PRSS50
CCDC12
PRSS45P
TAD borders:
H3K27Ac
iMac
CTCF
iMac
severe COVID-19 associated region
500 kb

Figure S5

Figure S5. Epigenomic landscape and 3D genome folding at the 3p21.31 COVID-19 risk locus in iMacs. (**a**) UCSC genome browser view of H3K27Ac and CTCF ChIP-Seq signal for the indicated cell types. Yellow shading indicates location of monocyte/macrophage-specific active chromatin regions (‘ACR1-3’). (**b**) Normalized gene expression levels (transcripts per million; TPM) for indicated 3p21.31 genes in iMacs. (**c**) Overview of A/B compartmentalization (derived from in-situ Hi-C) surrounding the 3p21.31 severe COVID-19 associated region (indicated by orange shading) in iMacs. PC1 values (at 100kb bin size) are shown as continuous profiles, with positive values (A compartment) in green and negative values (B compartment) in blue. (**d**) In-situ Hi-C contact map (at 5kb bin size) of the 3p21.31 COVID-19 risk locus in iMacs. TAD borders called are depicted as gray rectangles (called at 50kb resolution) and the topologically associating domain (TAD) encompassing most of the severe COVID-19 associated region (indicated by orange shading) is depicted by a dashed line. H3K27Ac and CTCF enrichment in iMacs is shown below.

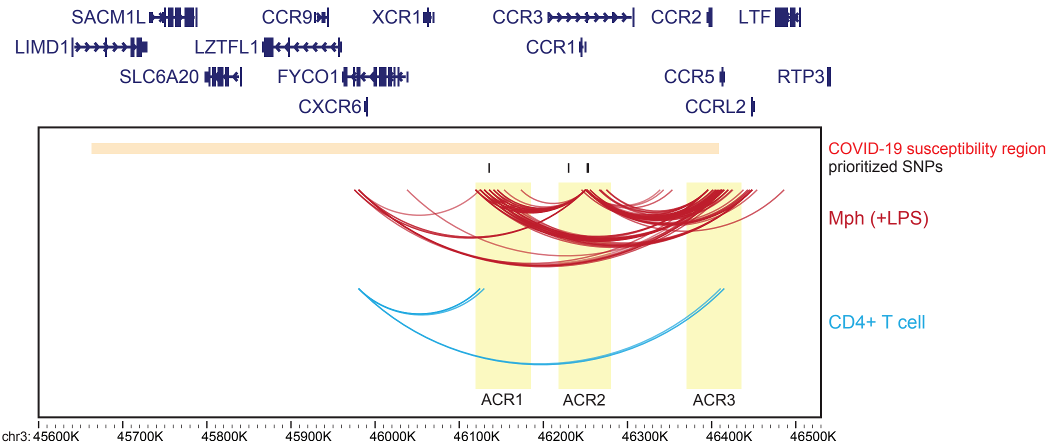
SACM1L
CCR9
XCR1
CCR3
CCR2
LTF
LIMD1
LZTFL1
CCR1
SLC6A20
FYCO1
CCR5
RTP3
CXCR6
CCRL2
COVID-19 susceptibility region
prioritized SNPs
Mph (+LPS)
CD4+ T cell
ACR1
ACR2
ACR3
chr3: 45600K
45700K
45800K
45900K
46000K
46100K
46200K
46300K
46400K
46500K

Figure S6

Figure S6. Chromatin interactions with prioritized 3p21.31 COVID-19 risk variants. Promoter capture Hi-C (PCHi-C) interactions[14] detected in monocyte-derived macrophages stimulated with LPS (red arcs) and CD4+ T cells (blue arcs). Active chromatin regions (ACR) 1, 2 and 3, as well as the GWAS-defined COVID-19 susceptibility region are indicated by yellow and orange shading, respectively. Interactions were only shown if at least one anchor connected to an ACR but irrespective of interaction span. Interactions connecting an ACR with sequences outside the depicted region were not detected. The 4 prioritized SNPs (located in ACR1 and ACR2) are shown on top.

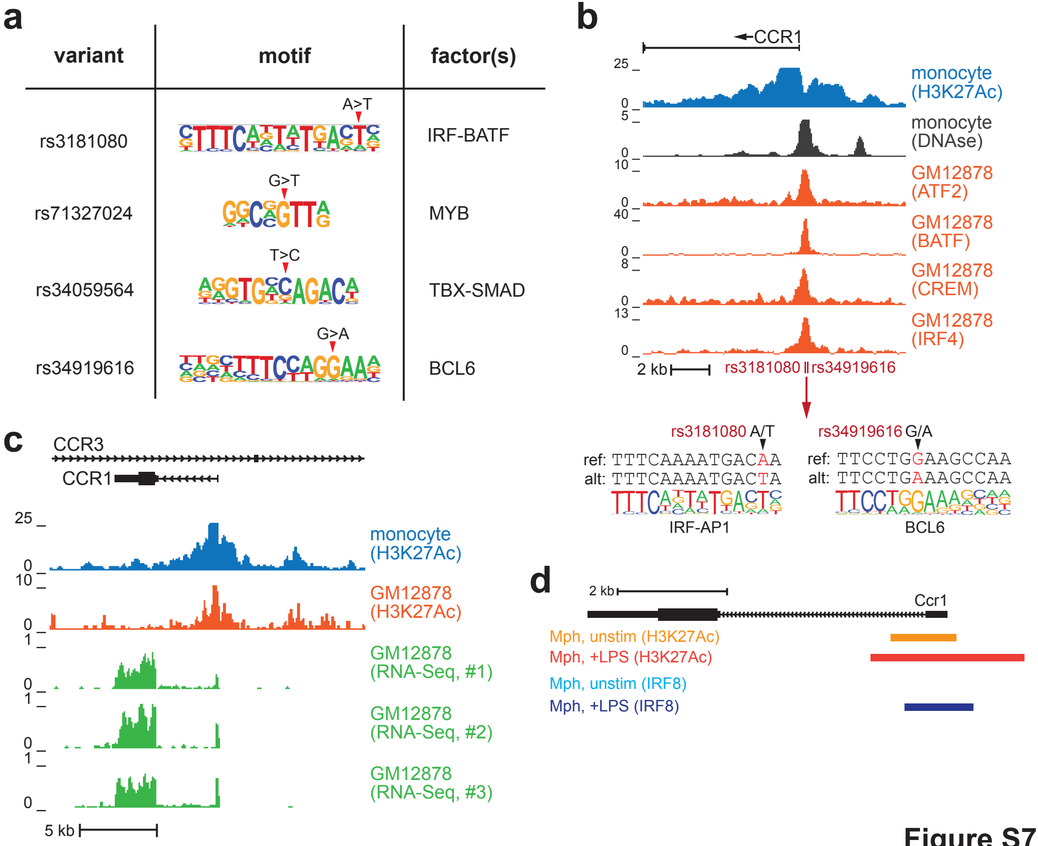
a
variant
motif
factor(s)
rs3181080
A>T
IRF-BATF
rs71327024
G>T
MYB
rs34059564
T>C
TBX-SMAD
rs34919616
G>A
BCL6
b
CCR1
monocyte (H3K27Ac)
monocyte (DNAse)
GM12878 (ATF2)
GM12878 (BATF)
GM12878 (CREM)
GM12878 (IRF4)
2 kb
rs3181080
rs34919616
rs3181080 A/T
rs34919616 G/A
ref: TTTCAAAATGACAA
alt: TTTCAAAATGACTA
ref: TTCCTGGAAGCCAA
alt: TTCCTGAAAGCCAA
IRF-AP1
BCL6
c
CCR3
CCR1
monocyte (H3K27Ac)
GM12878 (H3K27Ac)
GM12878 (RNA-Seq, #1)
GM12878 (RNA-Seq, #2)
GM12878 (RNA-Seq, #3)
5 kb
d
2 kb
Ccr1
Mph, unstim (H3K27Ac)
Mph, +LPS (H3K27Ac)
Mph, unstim (IRF8)
Mph, +LPS (IRF8)

Figure S7

Figure S7. 3p21.31 COVID-19 risk variants disrupt putative transcription factor binding sites. (**a**) Transcription factor binding motifs overlapping with four candidate causal SNPs (see Fig.2e). Single nucleotide substitutions are indicated above the motif logos. (**b**) Zoom-in view of the CCR1 promoter (top) showing H3K27Ac, chromatin accessibility (DNAse-Seq) and binding of AP1 (i.e. ATF2, BATF, CREM) or IRF4 transcription factors in the indicated cells (ENCODE database) overlapping two candidate causal SNPs. Binding motifs modified by the variants are indicated (bottom). Rs3181080 optimizes an IRF-AP1 composite motif (A>T), while rs34919616 disrupts a BCL6 motif (G>A). (**c**) UCSC genome browser view of the CCR1 gene (located inside an intron of CCR3) showing H3K27Ac ChIP-Seq and RNA-Seq data tracks for monocytes and GM12878 lymphoblastoid cells (ENCODE database). (**d**) UCSC genome browser view of the mouse Ccr1 gene. Locations of ChIP-Seq peaks for H3K27Ac and IRF8 detected in unstimulated or lipopolysaccharide (LPS) stimulated bone marrow-derived macrophages[24] are indicated by coloured rectangles.
