# Supplementary material for: Severe COVID-19-associated variants linked to chemokine receptor gene control in monocytes and macrophages
Source: Genome Biol. 2022 Apr 14;23:96. doi: 10.1186/s13059-022-02669-z (PMC9009160; doi:10.1186/s13059-022-02669-z)
Supplement: Supplementary file 3 — Additional file 3. Review history. [file 13059_2022_2669_MOESM3_ESM.docx]

Review History

**First round of review**

**Reviewer 1**

**Are you able to assess all statistics in the manuscript, including the appropriateness of statistical tests used?** Yes, and I have assessed the statistics in my report.

**Comments to author:**

The methods of the study are well described and appropriate to the achieved aims. In addition there are sufficient details to allow replication.The works has broad interest and represent an advance in the field, although the advance that it implicates is modest. In fact the implication of the 3p21.31 locus (i), monocytes and macrophages (ii) and chemokine receptors (iii) with COVID-19 is not completely novel.

This paper summarizes a purely descriptive study based on publicly available datasets and computational methods. In fact, functional validation (e.g., using iMacs to edit the lead and the prioritize variants with potential biological significance) will significantly increase the impact of the paper and these reaffirm the made conclusions. This experimental approach will not only validate the findings, but will also allow to detect distal alterations. For instance the prioritize variants with potential biological significance may regulate genes out of the susceptibility regions. In fact the authors claim that the leading SNP is not involved in significant interactions in blood cells, but maybe this result is a effect of the PHCi-C limited resolution (it is not possible to detect significant interactions between proximal sites)

**Reviewer 2**

**Are you able to assess all statistics in the manuscript, including the appropriateness of statistical tests used?** Yes, and I have assessed the statistics in my report.

**Comments to author:**

In Stikker et al., study, the authors leveraged several epigenetic datasets obtained public datasets to dissect the pathogenic mechanisms underlying the chr3p21.31 COVID-19 locus. Overall, the manuscript was well structurally organized, and the evidence is compelling.
In addition, there are multiple major issues which needs to be addressed before re-submission next time.
1. The risk variants are associated with severe COVID-19 (hospitalized), but not mild COVID-19. How the authors explain the CCR cluster would confer risk for severe COVID-19, but not mild COVID-19?
2. The majority of risk SNPs are within strong LD among each other. The authors used epigenetic datasets to fine map the causal SNPs, which is not enough. Other methods, such as statistical fine mapping using either European populations or mixed populations, are also required. The authors could also observe that the SNPs which are colocalized at the ACR regions (especially ACR3) show less stronger association with severe COVID-19 than the lead SNPs. In addition, the conclusion is different from both Prof. Rao (STTT, 2021) study. Is there any possibility that the variants at the ACR regions may be indeed functional, but may not causal for severe COVID-19.
3. Overall, if the authors can add extra experimental validations in COVID-19 mice, the conclusion might be greatly strengthened.

**GBIO-D-21-00912 Stikker et al. – Author response**

**General statement**

We want to thank both reviewers for taking interest in our study and providing constructive feedback, which we believe has further improved the quality of our study. Please find below our detailed responses to these comments. Resulting modifications to the manuscript text have been highlighted in yellow to facilitate reviewing.

**Reviewer #1**

*The methods of the study are well described and appropriate to the achieved aims. In addition there are sufficient details to allow replication. The works has broad interest and represent an advance in the field, although the advance that it implicates is modest. In fact the implication of the 3p21.31 locus (i), monocytes and macrophages (ii) and chemokine receptors (iii) with  COVID-19 is not completely novel.*

**Response:** We appreciate the positive assessment of our methodology and the interest in our findings. Whereas 3p21.31, monocytes/macrophages and chemokine receptor signaling have indeed been previously linked to COVID-19, our study is the first to provide compelling evidence that 3p21.31 COVID-19-associated variants are linked to altered CCR gene expression in monocytes or macrophages based on integrated analysis of GWAS, epigenomics and eQTL data. Importantly, our acute degron-mediated protein depletion experiments identified a novel molecular mechanism for CTCF-mediated transcriptional control of the 3p21.31 CCR gene cluster.

*This paper summarizes a purely descriptive study based on publicly available datasets and computational methods. In fact, functional validation (e.g., using iMacs to edit the lead and the prioritize variants with potential biological significance) will significantly increase the impact of the paper and these reaffirm the made conclusions.*

**Response:** We refrained from conducting functional validation experiments because of the notorious difficulties associated with trying to quantify the impact of putative causal SNPs in non-coding regions - especially when dealing with loci containing multiple independent significant SNPs, strong LD structure and highly complex gene regulatory landscapes (PMID: 32477401 & PMID: 25363779). The 3p21.31 region is a prime example of such a locus, with 20 independent significant SNPs that are in high LD (r^2^>0.8) with nearly 1000 SNPs, posing a major challenge for causal variant identification. Additionally, the multiple CCR genes are co-regulated by distal enhancers within the context of a 3D chromatin hub (see our combined data in **Fig.1**) - a situation that is difficult (if not impossible) to accurately mimic in small non-chromatinized reporter plasmid-based assays often used for validation purposes. Whereas genome-editing of endogenous loci can test variants in their native chromatin context, homology-directed repair (HDR) strategies required for editing are often very inefficient (PMID: 24606144) and therefore practically infeasible to conduct for more than a few isolated variants. Nevertheless, in an attempt to satisfy the reviewer’s recommendation we performed two series of experiments (involving our expert collaborators from Marseille), which are described in detail below and in **Reviewer Fig.1**. In line with our expectations, these efforts unfortunately did not yield any conclusive evidence. Given 1) the considerable investment in time and resources required to continue these experiments, 2) their inherent low chance of success and 3) the level of evidence already presented in the current manuscript (*designated by Reviewer #2 as ‘compelling’*) we feel that further pursuing of this path goes beyond the scope of this manuscript and would prevent the timely dissemination of our findings to a highly active COVID-19 research community.

**Reviewer Figure 1. (a)** CRISPR-Cas9 genome-editing strategy used. Homology-directed repair (HDR)-mediated editing of the THP-1 human monocyte cell line was chosen to introduce the indicated two prioritized CCR1 promoter COVID-19 risk variants. THP-1 cells were electroporated with two Cas9-gRNA ribonucleoprotein (RNP) complexes, a 200bp single-stranded repair template containing both risk variants and HDR enhancer solution (Integrated DNA Technologies). Single clones were picked, expanded and screened for the presence of risk alleles using PCR. **(b)** Luciferase reporter assay strategy used. The human CCR1 promoter (based on RNA Polymerase II ChIP-Seq signal enrichment) was cloned into a pGL3 luciferase reporter vector (Promega). Site-directed mutagenesis (Q5 kit, New England Biolabs) was used to engineer plasmids with all combinations of the CCR1 promoter variants. ACR1 and ACR2 putative enhancer elements representing DNAse-Seq peaks containing the rs71327024 and rs34059564 COVID-19 risk variants (major alleles), respectively, were cloned upstream of the CCR1 promoter. Site-direct mutagenesis was used to engineer plasmids with the indicated combinations of CCR1 promoter and ACR enhancer variants. Sanger sequencing was used to verify the sequence identity of all plasmids used. Plasmids were electroporated in U-937 (stimulated with PMA overnight) or iMac (at day 3 of transdifferentiation) myeloid cells and luciferase signals were measured after overnight culture. A fixed amount of Renilla-luciferase control plasmid was co-transfected to control for technical variation across individual reactions.

**- Approach 1:** We aimed to use CRISPR-Cas9 genome editing to generate myeloid cells carrying prioritized variants. We devised a strategy in which sgRNAs would create double-strand breaks in close vicinity of the two prioritized CCR1 promoter SNP locations (rs3181080 and rs34919616) in the THP-1 monocyte cell line (see **Reviewer Fig.1a**). This cell line is homozygous for the major alleles of both SNPs and harbors an active 3p21.31 CCR gene cluster. By providing HDR templates carrying the rs3181080/rs34919616 minor risk alleles, we attempted to create isogenic THP-1 lines carrying 0, 1 or 2 copies of the CCR1 promoter risk alleles. Unfortunately, testing multiple sgRNAs and screening hundreds of clonally expanded lines did not yield a THP-1 line carrying a risk allele, despite direct electroporation of Cas9-sgRNA ribonucleoprotein (RNP) complexes alongside an HDR enhancer (Integrated DNA Technologies). Due to the apparent inefficiency of HDR at this region we decided to abandon this approach and try an alternative strategy.

**- Approach 2:** We constructed 12 reporter plasmids to test the impact of genetic variants on CCR1 promoter/enhancer activity in myeloid cells (see **Reviewer Fig.1b**). We cloned the CCR1 promoter fragment carrying the major rs3181080/rs34919616 alleles in front of a luciferase reporter gene and measured well detectable promoter activity (i.e. luciferase signal) in U-937 myeloid cells, which also harbor an active 3p21.31 CCR cluster (PMID: 29746559) and showed good transfection efficiencies (~65%). ACR1 and ACR2 putative enhancer elements (containing prioritized risk variants) were cloned upstream of the CCR1 promoter to mimic regulation of CCR1 by ACR enhancers. For the ACR2 enhancer tested, this resulted in a 2.91 fold increase in CCR1 promoter activity (P=0.011, unpaired t-test). Site-directed mutagenesis was then used to construct vectors that carry the minor allele risk variants. Comparisons of CCR1 promoter activity in physiologically relevant combinations based on moderate-to-high LD (r^2^=0.4-1.0) in which the prioritized SNPs reside (i.e. promoter or promoter+ACR1/2, all major alleles vs. all minor alleles) did not reveal statistically significant changes in luciferase signal (P>0.2, unpaired t-test; similar results were obtained with iMac cells). However, we reckon that the lack of effect is not very surprising given the artificial nature of reporter assays in light of the considerable regulatory complexity of the the 3p21.31 CCR gene cluster (discussed above).

*This experimental approach will not only validate the findings, but will also allow to detect distal alterations. For instance the prioritize variants with potential biological significance may regulate genes out of the susceptibility regions.*

**Response:** The reviewer raises a valid point regarding potential distal alterations. In our manuscript, **Fig.1e** and **Fig.S4** show that the CCR1 promoter, harbouring the rs3181080 and rs34919616 prioritized variants, is not engaged in any significant interactions with sites outside the susceptibility region, with the exception of the CCRL2 gene just downstream of the region. To test whether the two prioritized enhancer variants may regulate genes (far) outside the susceptibility regions, we further mined PCHi-C data. As shown in **Reviewer Fig.2**, the same conclusion holds true for all four prioritized variants: the only noteworthy interactions outside the susceptibility region are just downstream with CCRL2, encoding a chemokine receptor also implicated in myeloid cell migration (PMID: 33363177). Importantly, no significant interactions were detected with sequences beyond the CCR gene cluster. These data and observations have now been added to the revised manuscript (new **Fig.S6** and **page 3-4**, **line 135-136**).

**Reviewer Figure 2.** Promoter capture Hi-C (PCHi-C) interactions detected in monocyte-derived macrophages stimulated with LPS (red arcs) and CD4+ T cells (blue arcs). Active chromatin regions (ACR) 1, 2 and 3, as well as the GWAS-defined COVID-19 susceptibility region are indicated by yellow and orange shading, respectively. Interactions were only shown if at least one anchor connected to an ACR but irrespective of interaction span. Interactions connecting an ACR with sequences outside the depicted region were not detected. The 4 prioritized SNPs (located in ACR1 and ACR2) are shown on top.

*In fact the authors claim that the leading SNP is not involved in significant interactions in blood cells, but maybe this result is a effect of the PHCi-C limited resolution (it is not possible to detect significant interactions between proximal sites).*

**Response:** We fully agree that the PCHi-C data we used (based on HindIII digestion) has limited capacity to detect very proximal interactions (i.e. within 20kb of the viewpoint), a technical limitation that we now mention in the paper (**page 2**, **line 85-86**). Of note, a recent study by Downes et al. supports our conclusion that the lead SNP region is unlikely to disrupt gene regulatory mechanisms in immune and other hematopoietic cells (PMID: 34737427). We now discuss the Downes et al. study in the context of our own findings (**page 4**, **line 173-180**); also see our response to comments from Reviewer #2 below.

**Reviewer #2**

*In Stikker et al., study, the authors leveraged several epigenetic datasets obtained public datasets to dissect the pathogenic mechanisms underlying the chr3p21.31 COVID-19 locus. Overall, the manuscript was well structurally organized, and the evidence is compelling.*

**Response:** We thank the reviewer for these positive remarks on manuscript organization and the strength of the evidence presented.

*In addition, there are multiple major issues which needs to be addressed before re-submission next time.
1. The risk variants are associated with severe COVID-19 (hospitalized), but not mild COVID-19. How the authors explain the CCR cluster would confer risk for severe COVID-19, but not mild COVID-19?*

**Response:** We only chose to show the data in Figure S1B to illustrate that the 3p21.31 locus exhibits a (somewhat) stronger association with severe/hospitalized COVID-19 than non-hospitalized COVID-19. However, we did not intend to argue that the 3p21.31 locus is not associated at all with mild COVID-19. In fact, 3p21.31 also confers risk for mild COVID-19 (see the latest GWAS data at <https://www.covid19hg.org/results/r6/> or in PMID: 34237774). We have modified the text to clarify this (**page 2**, **line 50-52**). We speculate that the stronger association with hospitalized COVID-19 may be due to increased CCR expression in monocytes being more important for disease severity than for susceptibility to infection.

*2. The majority of risk SNPs are within strong LD among each other. The authors used epigenetic datasets to fine map the causal SNPs, which is not enough. Other methods, such as statistical fine mapping using either European populations or mixed populations, are also required.*

**Response:** We fully agree and that is why we used the Functional Mapping and Annotation of Genome-wide Association Studies platform (FUMA), which employs a heuristic fine-mapping approach (PMID: 29184056). FUMA computes population-specific LD structure to identify independent significant SNPs (*r^2^*<0.6 with lead SNP) and imputes LD SNPs using the 1000 Genomes reference panel to identify genomic risk loci. Only after this statistical fine-mapping by FUMA we used epigenetics data for further refinement and prioritization.

We acknowledge that analysis of different ethnic populations could be helpful to further refine our variant prioritization strategy, as was recently shown for the OAS1 locus by leveraging weaker LD in African compared to European individuals (PMID: 33791713). However, in contrast to the OAS1 locus on chr12, the 3p21.31 locus also shows large LD blocks in an African population (**Reviewer Fig.3**), offering few opportunities for refinement - also when considering the severe lack of available COVID-19 GWAS data from non-Europeans (see PMID: 33888907 & 34237774).

**Reviewer Figure 3.** FUMA regional plots of 12q24.13 (containing OAS1; panel **a**) and 3p21.31 (containing the CCR cluster; panel **b**) using a European (top) or African (bottom) reference panel (1000 Genomes Phase 3) to compute linkage disequilibrium (r^2^ value).

*The authors could also observe that the SNPs which are colocalized at the ACR regions (especially ACR3) show less stronger association with severe COVID-19 than the lead SNPs. In addition, the conclusion is different from both Prof. Rao (STTT, 2021) study. Is there any possibility that the variants at the  ACR regions may be indeed functional, but may not causal for severe COVID-19.*

**Response:** Variants in the ACR regions indeed show a less strong association with risk to develop severe COVID-19 than the lead SNP. However, ACR SNPs are in very high LD (r^2^>0.89) with independent significant SNPs in the region and show highly significant GWAS P values < 10^-10^, justifying their consideration as putative causal SNPs. Nevertheless, we acknowledge that variants and genes closer to the lead SNP could also be causally involved in conferring risk for severe COVID-19. Hence, we believe strategies aimed at investigating mechanisms around the LZTFL1 lead region to be very important, including the study led by Prof. Rao (cited in our original manuscript) but also recent work by Downes et al. (PMID: 34737427). As we explain in our discussion section (**page 4**, **line 169-182**), our analyses by no means rule out other causal variants and mechanisms affecting COVID-19 pathophysiology other than through CCR gene regulation in innate immune cells. Multiple distinct biological pathways active in different cell types could underlie the 3p21.31 association, perhaps even involving more than one independent genetic association (see PMID: 34237774 for supporting evidence). For example, upregulation of LZTFL1 by rs17713054 in lung epithelial cells may promote viral entry (PMID: 34737427), whereas upregulation of CCR1 by rs3181080 in monocytes could exacerbate pulmonary inflammation and lead to more severe disease (our study).

*3. Overall, if the authors can add extra experimental validations in COVID-19 mice, the conclusion might be greatly strengthened.*

**Response:** We agree with the reviewer that experimental validation using a mouse model for COVID-19 pathophysiology represents a valuable next chapter in further dissecting the biological mechanisms underlying the 3p21.31 risk locus. However, such studies represent a massive investment in time and resources, which could easily take several years of work (i.e. mouse strain import, animal breeding, model optimization and implementation, conducting various experiments). Hence, we feel that *in vivo* experiments go beyond the scope of this manuscript and would also prevent timely dissemination of our findings, which are immediately relevant for the COVID-19 research community.

**Second round of review**

**Reviewer 1**

I want thank the effort of the authors to address the suggestions. I am aware of the difficulties regarding the functional validation. For this reason, although this data could significantly improve the quality of the data, the impact of the manuscript itself is good. Thus, I recommend to publish the manuscript.

**Reviewer 2**

The authors have addressed all my comments. I don't have any more comments.
